# Sleep Environment and Insomnia in Elderly Persons Living at Home

**DOI:** 10.1155/2018/8053696

**Published:** 2018-09-27

**Authors:** Jonathan Desaulniers, Sophie Desjardins, Sylvie Lapierre, Alain Desgagné

**Affiliations:** ^1^Department of Psychology, Université du Québec à Trois-Rivières, C.P. 500, Trois-Rivières, Québec, Canada G9A 5H7; ^2^Department of Mathematics, Université du Québec à Montréal, C.P. 8888, Succursale Centre-ville, Montréal, Québec, Canada H3C 3P8

## Abstract

The aim of this study was to draw a portrait of the sleep environment of elderly persons living in private households and to determine its relationship with the presence of insomnia. A sample of 599 individuals aged 70 years and older responded to questions about the comfort of their pillow and mattress and the noise level and brightness of their bedroom at night and in the morning. They were also asked whether or not they shared their bed or bedroom with a sleep partner. The Insomnia Severity Index was used to assess insomnia severity. Over 40% of the study participants were using a pillow that was not very comfortable, and almost 30% said that their bedroom was not completely quiet. Binomial logistic regression results revealed that two variables were significantly associated with insomnia symptoms: a pillow rated as moderately comfortable to very uncomfortable and a bedroom that was not completely quiet. No other sleep environment characteristics considered in this study were associated with the risk of insomnia. These results indicate that a nonnegligible proportion of the elderly population endures a suboptimal sleep environment. Although it is difficult to predict the real impact of changes to the sleep environment, this study supports the proposal that simple, minor changes to the bedroom can promote sleep in the elderly.

## 1. Introduction

The frequency of self-reported sleep problems increases with age. Whereas 40% of individuals who consult a general physician complain of significant sleep problems, the proportion rises to 63% for individuals aged 60 years and over, and to 67% if they are living in a long-term care residence [1–3]. As life expectancies rise in the vast majority of industrialized countries [4, 5], sleep problems will become a growing concern.

Although certain physiological changes that affect sleep quality are considered normal with increasing age, cognitive behavioral therapy (CBT) is used as a standard first-line treatment to reduce many sleep-related symptoms in the elderly [6, 7]. Such treatment generally involves recommendations for good sleep hygiene, including changes to the sleep environment. Elderly persons are at greater risk for nocturnal awakenings and sleep fragmentation compared with the general population [8]. Consequently, they are at greater risk for being affected by an inadequate sleep environment, namely, a bedroom that is insufficiently quiet, dark, or comfortable.

Because the brain processes auditory stimuli in both the awake and asleep state, sleep quality can be affected by auditory pollution [9]. When the noise level is within the range from 40 to 55 dB or approximately the volume of light road traffic, people generally try to adapt their environment to preserve adequate sleep quality. At noise levels above 55 dB or approximately the volume of two people having a conversation, sleep problems become more frequent, depending on whether hearing acuity is preserved [10].

Exposure to nocturnal light is another risk factor for sleep problems. Nocturnal light inhibits the secretion of melatonin, a hormone involved in sleep regulation [11, 12]. Moreover, in the elderly, nocturnal light can promote the development of depressive symptoms [13], raise blood pressure [14], and contribute to the development of carotid stenosis [15]. Conditions such as advanced or delayed sleep phase disorder can also impair sleep quality in this population [16].

Pillow comfort can also affect sleep quality [17] because a properly designed pillow allows greater surface contact with the neck, for a more even distribution of pressure on the cervical (neck) muscles [18]. Although the shape, thickness, and material of commercially available pillows vary, consumers continue to choose based largely on personal preferences [19]. However, for the general population, feather pillows may be less performant than those made of other materials [17, 20]. Considering that the prevalence of chronic pain ranged from 23.9 to 31.3% in Canadians aged 65 years and older from 1994 to 2008 [21], studies have examined the use of pillows designed to alleviate cervical pain. Although the specifications for these pillows vary across studies, a simple change of pillow can improve the sleep of elderly persons [22, 23]. Still, the findings of the most recent meta-analysis on this topic do not allow recommending the use of a cervical pillow to improve sleep quality in a population presenting chronic cervical pain [24].

Mattress comfort can also affect sleep quality by making it easier to change position during the night to prevent muscular stiffness [25]. Studies conducted in adults with chronic pain underscore the benefits of a moderately firm mattress to optimize sleep in this population, independently of age [26, 27]. In addition, variations in weight and height can affect perceptions of comfort [28]. However, the current state of knowledge does not allow specifying the mattress characteristics that are likely to promote sleep in the elderly [29–31].

Given the prevalence of sleep problems in the elderly and the difficulty of access to healthcare specialists, including lengthy wait times, researchers should delve more deeply into the specific contribution of the sleep environment. The results could help empower older individuals to manage sleep problems with no demonstrable organic cause. Currently, the individual influences of environmental factors that could improve sleep quality remain unknown [32], and the same goes for the typical sleep environment of elderly persons. In order to fill this research gap, the present study aims, on the one hand, to draw a portrait of the sleep environment of elderly persons, and on the other hand, to determine whether the presence (or not) of a partner in the same bed or bedroom, the noise and brightness of the bedroom, and pillow and mattress comfort allow distinguishing between elderly persons who present insomnia and those who do not.

## 2. Materials and Methods

The participants initially took part in the longitudinal Québec Survey on Seniors' Health (Enquête sur la santé des aînés), which addressed a representative sample (*n*=2798) of elderly persons living in the community (i.e., in private households) [33]. These individuals were recontacted by mail and invited to participate in the present study. Once the study objective was explained and the participants had given their informed consent to participate, they filled out questionnaires on sociodemographic characteristics, sleep environment characteristics, and sleep quality. The participation rate for the present study was over 20%. This rate is satisfactory given that it can be explained not only by refusal, but also by mortality, disability, and loss of contact. This study was approved by the Research Ethics Committees of the Université du Québec à Trois-Rivières (UQTR) and the Institut universitaire de gériatrie de l'Université de Sherbrooke (IUGS).

Insomnia symptoms were assessed with the Insomnia Severity Index (ISI) [34, 35]. This questionnaire contains seven questions on participants' perceptions of the type, severity, and impact of insomnia within the past month, for a total score ranging from 0 to 28. A score from 0 to 7 indicates absence of insomnia, from 8 to 14 mild insomnia, from 15 to 21 moderate insomnia, and 22 and higher severe insomnia. Cronbach's alpha for the questionnaire items varies from 0.74 to 0.78 [34]. Content validity was demonstrated by principle component analysis, which identified three components: impact of insomnia, insomnia severity, and satisfaction with sleep. Despite some overlap between these components, they allow capturing an insomnia diagnosis as defined by the DSM-IV [34]. An ISI score of 14 demonstrates 94% sensitivity and 94% specificity to distinguish individuals presenting a diagnosis of primary insomnia from good sleepers in a control group. The psychometric properties of the questionnaire have been demonstrated for the English version and the French translated version [34, 35].

The sleep environment was explored with a set of seven questions referring to the past month, as follows: “How comfortable was your pillow?”; “How comfortable was your mattress?”; “What was the level of noise in your bedroom at night?”; “What was the level of noise in your bedroom in the morning?”; “How bright was your bedroom at night?”; and “How bright was your bedroom in the morning?” Responses were rated on a four-point Likert scale. For pillow and mattress comfort, the response choices were “Very uncomfortable,” “Not very comfortable,” “Moderately comfortable,” and “Very comfortable,” with 1 for lowest and 4 for greatest comfort. Similarly, for noise level, the choices were “Very loud,” “Moderately loud,” “Not very quiet,” and “Completely quiet,” and for brightness, the choices were “Very bright,” “Moderately bright,” “Moderately dark,” and “Very dark.” Participants were also asked if a partner shared the same bed or same bedroom: “Did you share your bed or your bedroom with a partner?” The four response choices were: “I did not share my bed or my bedroom with a partner,” “My partner or roommate slept in a separate room,” “My partner slept in my bedroom but in a separate bed,” and “I slept in the same bed as my partner.”

First, descriptive analyses were performed. *T*-tests and Pearson's correlations were calculated to examine associations with the variables. Participants who scored a total of 8 or more on the ISI (*n*=245) were classified into the Insomnia group and those who scored less than 8 (*n*=343) were classified into the No-insomnia group. A binomial logistic regression followed by model adjustment of all the study variables were then performed to estimate the probability of belonging to the Insomnia group according to the sleep environment and sociodemographic variables. The assumptions of multicollinearity were met. The analyses were performed using SPSS Statistics 24 at a significance level of 5%.

## 3. Results

### 3.1. Participant Characteristics

Participants (*N*=599) were 79.3 years old on average (range = 70.0–94.6 years). All lived at home. Women accounted for 69.6% of the sample. Of the women, 3.1% were below normal weight, 41.2% normal weight, 34.8% overweight, and 20.9% obese. Of the men, 1.7% were underweight, 35.1% normal weight, 48.9% overweight, and 14.3% obese. Almost 40% of participants were married and lived as a couple. Only 24.1% had completed a university degree, and 42% of participants had an average annual income less than $25,000.

Table 1 presents the descriptive statistics according to the presence (or not) of insomnia. With respect to comfort, 52.8% of participants reported sleeping with a very comfortable pillow and 57.8% on a very comfortable mattress. The bedroom was quiet at night for 70.5% of participants and quiet for 62.4% in the morning. The bedroom was moderate to very dark for 8.7% of participants at night and in the morning for 55.2%. Almost 70% of participants slept alone in a separate bedroom.

### 3.2. Comparison of Participants with and without Insomnia

Elderly persons who slept with a very uncomfortable pillow or mattress experienced insomnia significantly more often than those who reported their pillow or mattress as from not very comfortable to very comfortable. Similarly, insomniac participants slept significantly more often in a bedroom that was not completely quiet, both at night and in the morning.  Table 2 presents the binomial logistic regression results. The adjusted model indicates that the probability of suffering from insomnia is twice as great when the pillow was rated from moderately comfortable to very uncomfortable and the bedroom was rated as not completely quiet at night. No other sleep environment characteristic was associated with a higher risk of insomnia while accounting for the other variables. The percentage of variance explained by the model is between 5.5% and 7.4%, suggesting that other variables not considered in this study would be more influential.

## 4. Discussion

Our results indicate that a nonnegligible proportion of the elderly population endures a suboptimal sleep environment. Two components are significantly associated with insomnia symptoms: a pillow rated as moderately comfortable to very uncomfortable and a bedroom that was rated as not completely quiet.

These findings highlight the fact that many elderly persons sleep with a pillow that is uncomfortable to some extent and that these individuals are more likely to be insomniacs. Almost half of the participants reported that their pillow was not very comfortable. Given the relatively affordable cost of a new pillow, we might wonder what would justify hanging on to an uncomfortable one. Perhaps the vast choice of materials, shapes, and sizes available on the market [19, 23] combined with a lack of guidelines for consumers [20] would discourage some elderly persons from making a change. In fact, the study participants appeared to have had trouble finding a pillow that met their needs. These results support studies that have found that many people continue using an uncomfortable pillow, even when they wake up in pain [36]. Further studies are needed to understand the factors that influence pillow choice, as well as the pillow features that can provide comfort, diminish pain upon awakening, and promote good sleep quality in the elderly. At the same time, many cohort studies have found evidence to support a reciprocal relationship between sleep problems and chronic pain [37]. Because chronic pain was not measured in the present study, it is possible that some results could be partly attributable to the absence of control for pain in this sample. Given that the cervical region grows more vulnerable with age, it becomes all the more important to choose a good pillow [25].

Second, our results add to the evidence on increased symptoms of insomnia in the elderly when exposed to nocturnal noise [9, 38–41]. Almost 30% of our participants reported that they slept in a room that was not completely quiet at night. It is probable that some elderly persons ignore or play down the consequences of nocturnal noise. Because the elderly are at greater risk for nocturnal awakenings and sleep fragmentation than the general population [8], they would be at greater risk for suffering the consequences of a loud sleep environment, insofar as their hearing acuity is preserved. However, [42] demonstrated that simply using earplugs reduced noise by 7 to 12 dB, thereby promoting deep sleep. Furthermore, insulating the bedroom walls and replacing windows reduced noise by an average of 7 dB [43].

Over 40% of our participants reported that their mattress was not very comfortable. Despite the available information on sleep ergonomics, notably the importance of a good mattress to support the natural curvature of the spine, these findings indicate that insomnia was not associated with mattress discomfort. In view of the significant association found for pillow discomfort, this result is intriguing. It is possible that insomniac individuals tend to attribute certain symptoms to pillow discomfort rather than mattress discomfort. Because changes in position during nocturnal awakenings result in postural changes that affect the neck more than other parts of the body, the mattress may be perceived as playing a lesser role. Furthermore, the cost of a new mattress combined with the physical effort required to manoeuver it onto the bed could also influence how elderly persons perceive its impact on the sleep environment. This cognitive bias could steer complaints toward a factor that is more easily controlled, which could explain why the pillow was blamed more readily than the mattress. Although perceptions of mattress comfort could also be influenced by individual morphology, our finding showed no significant association between body mass index (BMI) and insomnia.

Approximately 12% of participants reported sleeping in a bedroom that was moderately or very bright at night. Although this proportion is small, making a room darker is a simple and affordable fix, and the reluctance to do so is puzzling. The normal deterioration of vision and the higher risk of falling in the elderly might threaten feelings of safety, whence the desire to keep the room at least dimly lighted at night. However, our results contradict studies that demonstrated a significant association between insomnia and exposure to nocturnal light in elderly populations [44, 45]. Although exposure to nocturnal light disrupts melatonin secretion and can consequently delay sleep, there is insufficient evidence in the literature to support a direct association between exposure to nocturnal light and insomnia. Nevertheless, it would be advisable to limit nocturnal light exposure in order to maintain a balanced circadian rhythm and prevent the development of disorders associated with an asynchronous circadian rhythm.

Compared with loud noise in the bedroom at night, loud noise in the morning was not associated with higher risk of insomnia. This could be explained by the systematic finding that elderly persons wake up earlier than the general population [46]. It is therefore possible that morning noise would be interpreted as time to get up. Rather than considering the noise as a problem, some individuals would simply get up and begin their day.

Almost 70% of participants slept alone in a separate bedroom, even though 44% lived as a couple. This practice could be partly explained by the high prevalence of unsatisfactory unions in the elderly population found in some studies [47]. The fragmented sleep of one member of the couple would probably lead to separate sleeping arrangements to preserve the sleep quality of the unaffected partner. In the population of middle-aged men, 17.5% snore loudly, and this proportion rises with age [48] to 33% of men and 19% of women aged 65 years and over [49]. It is reasonable to believe that some partners would prefer to sleep in a separate room to escape loud snoring. Separate rooms could also be explained by certain physical conditions, such as restless legs syndrome, which increases in prevalence with age and affects from 7 to 23% of individuals aged 40 years and over [50].

Among the limitations of this study, it is important to mention the impossibility of establishing a causal relationship between insomnia and either pillow comfort or nocturnal noise. Due to the cross-sectional study design, the relationships between the environmental factors and insomnia could be two directional. Thus, it is possible that a suboptimal sleep environment causes insomnia, which does not exclude the possibility that insomnia could lead to perceptions that certain aspects of the sleep environment are disturbing. The subjective measures used in this study would have been improved by the addition of objective measures to allow comparison with other studies. Considering that depression is an important risk factor for the development of sleep problems [51], and given the known association between these two conditions [52], it would have been relevant to assess the presence of depressive symptoms in our sample. In addition, pain assessments would have been useful to distinguish between insomnia that was primarily associated (or not) with physical pain. Hearing acuity could also have been determined, and earplug use could have been controlled for. These additional measures would allow further isolating the influence of sleep environment variables.

Despite these limitations, our results are based on a large sample of 600 elderly persons aged 70 years and over. All participants lived at home, which provided greater choice in arranging the sleep environment. The statistical analyses enabled assessing the contribution of each variable while taking into account the overall sleep environment, resulting in the identification of two sleep environment variables that were significantly associated with insomnia in our sample.

In a field that has received little attention, our results testify to the fact that a nonnegligible proportion of the elderly population endures a suboptimal sleep environment. The significant associations found between insomnia and pillow comfort as well as nocturnal noise point to future research avenues. To date, no studies to our knowledge have measured the impact of noise reduction at night on the sleep of elderly persons with insomnia. More studies are also needed to explore the impact of pillow replacement on elderly insomniacs. We do not know which factors contribute to the choice of a new pillow or the decision to continue using an uncomfortable pillow or mattress. Future studies could also look at the average age of pillows and mattresses used by elderly persons to determine the influence of wear and tear on insomnia. Although our study supports the research that demonstrates the harmful effect of nocturnal noise, more studies are needed to better target the consequences for sleep in the aging population. Similarly, the consequences of and the underlying reasons for the widespread practice of sleeping in a separate room remain unclear. Considering that it is preferable to use a combination of objective and subjective measures to examine sleep in the elderly [53], we recommend that future studies take advantage of a variety of available measures to gain a deeper understanding of the sleep experience in this population. Finally, pharmaceutical trials and CBT clinical trials should routinely measure or control for environmental factors when studies are done in the home. This environmental information might be used as covariates to improve signal detection of the primary intervention.

## 5. Conclusion

Although it is difficult to predict the actual impact of changes in the sleep environment of elderly persons, this study lends credibility to the hypothesis that simple, minor, and easily managed changes can promote sleep. Weighed against the minimal to nonexistent risks associated with such environmental changes, the potential benefits for the physical and psychological health of the elderly are substantial.

## Figures and Tables

**Table 1:** Descriptive statistics according to the presence or absence of insomnia.

Variable	Total	Insomnia	Absence of insomnia	*p*
Age	79.29 (4.87)	79.17 (4.68)	79.38 (5.01)	0.603
Sex				0.119
** **Male	179 (30.4)	66 (36.9)	113 (63.1)	
** **Female	409 (69.6)	179 (43.8)	230 (56.2)	
Body mass index (BMI)	26.21 (4.41)	26.08 (4.16)	26.31 (4.58)	0.553
Civil status				0.482
** **Living alone	330 (56.3)	141 (42.7)	189 (57.3)	
** **Living as a couple	256 (43.7)	102 (39.8)	154 (60.2)	
Highest education level				0.646
** **Zero to 12 years	338 (58.5)	140 (41.4)	198 (58.6)	
** **College to university degree	240 (41.5)	104 (43.4)	136 (56.7)	
Partner sleeps in the same bed or bedroom				0.306
** **Sleeps alone in the bedroom	394 (69.1)	168 (42.6)	226 (57.4)	
** **Partner sleeps in the same bedroom	176 (30.9)	67 (38.1)	109 (61.9)	
Pillow comfort				<0.001
** **Very uncomfortable to moderately comfortable	273 (47.2)	138 (50.5)	135 (49.5)	
** **Very comfortable	306 (52.8)	104 (34.0)	202 (66.0)	
Mattress comfort				0.023
** **Very uncomfortable to moderately comfortable	244 (42.2)	115 (47.1)	129 (52.9)	
** **Very comfortable	334 (57.8)	126 (37.7)	208 (62.3)	
Nocturnal bedroom noise				0.001
** **Very loud to not very loud	171 (29.5)	89 (52.0)	82 (48.0)	
** **Completely quiet	408 (70.5)	152 (37.3)	256 (62.7)	
Morning bedroom noise				0.030
** **Very loud to not very loud	216 (37.6)	102 (47.2)	114 (52.8)	
** **Completely quiet	358 (62.4)	136 (38.0)	222 (62.0)	
Bedroom brightness at night				0.667
** **Very bright to moderately bright	71 (12.3)	28 (39.4)	43 (60.6)	
** **Moderately dark to very dark	508 (87.7)	214 (42.1)	294 (57.9)	
Bedroom brightness in the morning				0.452
** **Very bright to moderately bright	259 (44.8)	104 (40.2)	155 (59.8)	
** **Moderately dark to very dark	319 (55.2)	138 (43.3)	181 (56.7)	

Data are presented as mean (standard deviation) or number of participants (percentage).

**Table 2:** Odds ratio and confidence intervals for the predictive variables for the presence or absence of insomnia.

Variables	Insomnia vs. absence of insomnia^a^	
	OR	CI 95 %

Age	1.03	0.99–1.07
Sex		
** **Male	1.44	0.94–2.21
** **Female	1.00	
Body mass index (BMI)	1.00	0.96–1.05
Civil status		
** **Living along	1.06	0.62–1.81
** **Living as a couple	1.00	
Highest education level		
** **Zero to 12 years	0.78	0.53–1.15
** **College to university degree	1.00	
Partner sleeps in the same bed or bedroom		
** **Sleeps alone in the bedroom	1.01	0.57–1.81
** **Partner sleeps in the same bedroom	1.00	
Pillow comfort		
** **Very uncomfortable to moderately comfortable	2.00^*∗∗*^	1.27–3.17
** **Very comfortable	1.00	
Mattress comfort		
** **Very uncomfortable to moderately comfortable	0.97	0.61–1.53
** **Very comfortable	1.00	
Nocturnal bedroom noise		
** **Very loud to not very loud	2.20^*∗∗*^	1.26–3.82
** **Completely quiet	1.00	
Morning bedroom noise		
** **Very loud to not very loud	0.70	0.41–1.18
** **Completely quiet	1.00	
Bedroom brightness at night		
** **Very bright to moderately bright	0.88	0.48–1.62
** **Moderately dark to very dark	1.00	
Bedroom brightness in the morning		
** **Very bright to moderately bright	0.95	0.64–1.42
** **Moderately dark to very dark	1.00	

^a^Reference group. OR, odds ratio; CI, confidence interval. ^*∗∗*^*p* < 0.01. Model *χ*^2^_(12)_ = 28.70, *p* < 0.004. *R*^2^ =  0.055 (Cox and Snell), 0.074 (Nagelkerke).

## Data Availability

The data used to support the findings of this study are available from the corresponding author upon request.
